# Square Wave Voltammetry of TNT at Gold Electrodes Modified with Self-Assembled Monolayers Containing Aromatic Structures

**DOI:** 10.1371/journal.pone.0115966

**Published:** 2014-12-30

**Authors:** Scott A. Trammell, Dan Zabetakis, Martin Moore, Jasenka Verbarg, David A. Stenger

**Affiliations:** Center for Bio/Molecular Science and Engineering, Naval Research Laboratory, Washington, DC, United States of America; Queen’s University at Kingston, Canada

## Abstract

Square wave voltammetry for the reduction of 2,4,6-trinitrotoluene (TNT) was measured in 100 mM potassium phosphate buffer (pH 8) at gold electrodes modified with self-assembled monolayers (SAMs) containing either an alkane thiol or aromatic ring thiol structures. At 15 Hz, the electrochemical sensitivity (µA/ppm) was similar for all SAMs tested. However, at 60 Hz, the SAMs containing aromatic structures had a greater sensitivity than the alkane thiol SAM. In fact, the alkane thiol SAM had a decrease in sensitivity at the higher frequency. When comparing the electrochemical response between simulations and experimental data, a general trend was observed in which most of the SAMs had similar heterogeneous rate constants within experimental error for the reduction of TNT. This most likely describes a rate limiting step for the reduction of TNT. However, in the case of the alkane SAM at higher frequency, the decrease in sensitivity suggests that the rate limiting step in this case may be electron tunneling through the SAM. Our results show that SAMs containing aromatic rings increased the sensitivity for the reduction of TNT when higher frequencies were employed and at the same time suppressed the electrochemical reduction of dissolved oxygen.

## Introduction

The electrochemical reduction of nitro-aromatics is a convenient detection method for common explosives such as TNT. Numerous studies have looked at the fundamental mechanism of electrochemical reduction, as well as more applied schemes to enhance the electrochemical detection for analytical applications [1]–[4]. Various electrode materials have been explored to enhance nitro-aromatic detection including activated carbon fiber electrodes and screen-printed carbon electrodes, but there are only a few reports on modified gold electrodes [5]–[7]. A common way to modify gold electrodes is by using the self-assembly of thiol-containing molecules from solution. Sulfur compounds adsorb spontaneously onto gold surfaces forming S–Au bonds making well packed and ordered monolayers. For electroanalytical application, the technique has recently been reviewed [8]. The molecules used in the creation of self-assembled monolayers (SAMs) typically contain a thiol for attachment to gold and a headgroup that is exposed to solution.

Square-wave voltammetry is an electroanalytical technique which diminishes non-faradaic charging currents that develop at the solution/electrode interface when employing potential sweeping techniques [9] and has been used for the electrochemical detection of nitro-aromatics [10]. For TNT detection at modified gold electrodes, a comparison study between hydrophobic and hydrophilic SAMs i.e. -CH_3_ or -OH terminal group-heads showed a greater response with the hydrophilic SAMs tested at 15 Hz using a rotated disk electrode [7]. Our goal in this study was to investigate the electrochemical response for the reduction of nitro-aromatics by modifying gold electrodes with molecules containing aromatic structures. We report on the comparison of three different self-assembled monolayers (SAMs), biphenyl-4-thiol (biphenyl), 4-(phenylethynyl)benzenethiol (OPE) and undecane-1-thiol (C11), using square wave voltammetry for the reduction of TNT.

**Figure 1:**
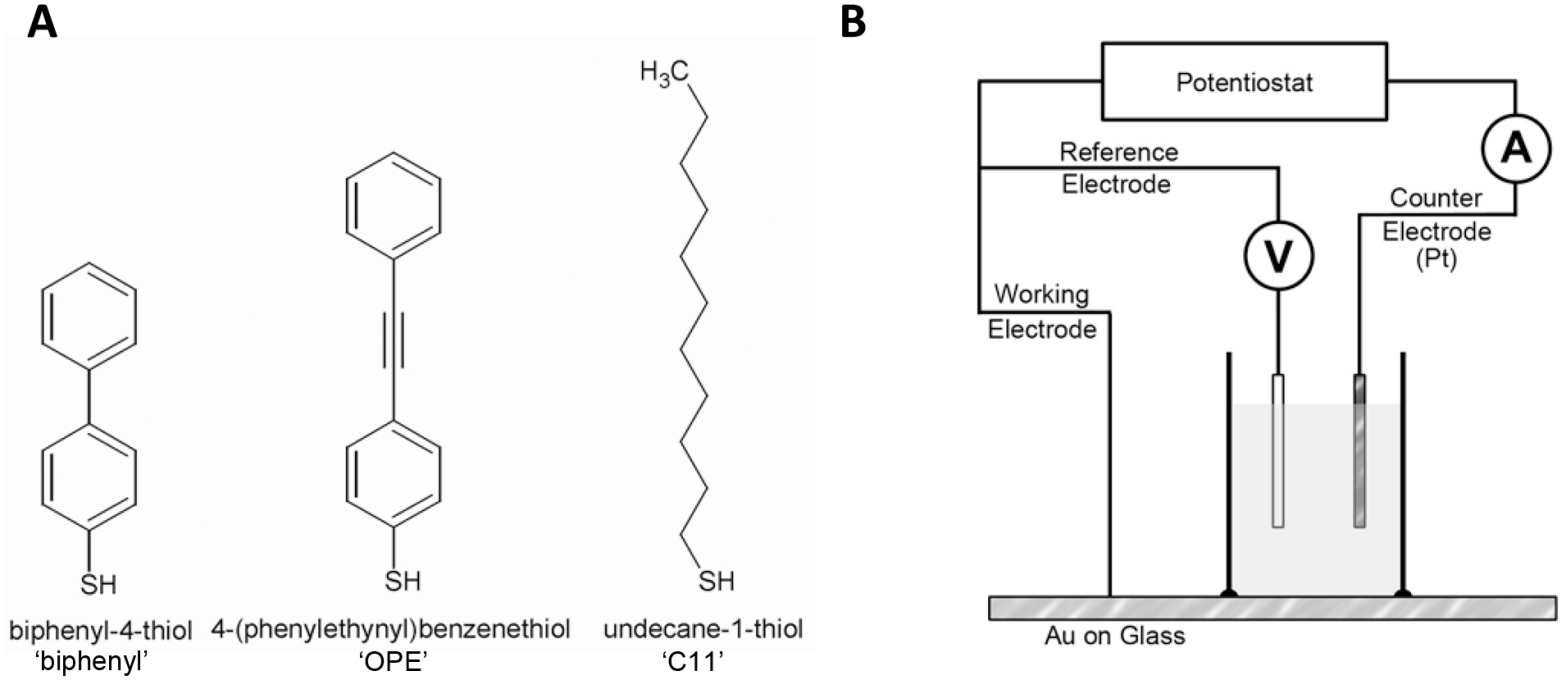
Experimental details. **A.** Chemical structures for biphenyl-4-thiol (Biphenyl), 4-(phenylethynyl)benzenethiol (OPE) and undecane-1-thiol (C11) used to from SAMs on gold electrodes. **B.** The electrochemical setup.

The structures of the molecules are shown in Fig. 1A. Two of the structures contain aromatic rings, which should promote electron tunneling across the SAMs [11], [12] on the modified electrode to the analyte in solution. The SAMs have been well characterized in the chemical literature [13]–[15]; however, the comparison between aromatic structures and alkane SAMs for their ability to modulate the electrochemical response of nitro-aromatics has not been explored. In this regard we show that SAMs containing aromatic rings yield a larger signal which is desirable when using square wave voltammetry as the detection technique in liquid chromatography systems [16], [17].

## Materials and Methods

### Materials

Undecane-1-thiol (C11), potassium phosphate monobasic, and the TNT standard in acetonitrile were purchased from Sigma-Aldrich and used as received. Biphenyl-4-thiol (biphenyl) and 4-(phenylethynyl)benzenethiol (OPE) were synthesized as reported in the chemical literature [18]–[20].

### Electrochemistry

Gold films on glass were purchased from Evaporated Metal Films. They were scored and cleaved into 1 cm×2.54 cm pieces. The gold films were then cleaned by argon plasma etch (15 min, 200 mTorr, 100 W R_f_) and immediately modified with a SAM from toluene solutions of SAM molecules at concentration of 1 mg/mL. After overnight exposure, the films were rinsed with fresh toluene and dried under nitrogen. Contact angle measurements were made on a Zisman-type contact angle goniometer. Electrochemical measurements were using a standard 3-electrode setup (Fig. 1B). The modified gold electrodes were mounted in an electrochemical cell with a defined surface area controlled by a rubber gasket. The parameters of the square voltammetry were - sample internal = 4 mV, amplitude = 25 mV and frequency = 15 or 60 Hz.

The coverage of the SAM molecules on the modified gold electrodes were calculated by integrating the desorption peak from the resulting cathodic stripping voltammetry performed in argon-saturated solutions using 0.5 M KOH buffer [21]. The double layer capacitance of each SAM was calculated from the CVs [9] measured at 0 V vs. Ag/AgCl in cyclic voltammograms recorded between 0.2 and −0.2 V vs. Ag/AgCl at scan rates of 100 and 200 mV/s in pH 8 buffer under Ar. TNT electrochemical measurements were recorded in air-saturated solutions with 100 mM potassium phosphate electrolyte adjusted to pH 8. All electrochemical measurements were made with a Ag/AgCl reference electrode and a Pt counter electrode performed using potentiostats model #440 or #660 from CH Instruments. Electrochemical simulations were performed using DigiElch version 7 (Gamry Instruments Inc.) and the details of the simulation have been include in supporting information and simulation parameters are shown in S1 Table in S1 Appendix. Square wave voltammetry data was exported from the CH Instruments software and reformatted for import into the simulation software.

## Results

### Electrode modification and characterization

Modification of the gold electrodes by the SAM molecules was accomplished by exposing the electrodes to solutions containing thiol molecules at a concentration of 1 mg/mL for an overnight incubation. After a thorough rinse and drying under nitrogen, the SAMs were characterized by contact angle measurements, surface coverage estimates, and double layer capacitance experiments. The data is listed in Table 1. The contact angles are typical of hydrophobic surfaces [15], [22]. The amount of surface coverage (nmoles/cm^2^) of each SAMs estimated from integrating the desorption peak from the cathodic stripping voltammetry (S1 Fig. in S1 Appendix). The double layer capacitance measured from cyclic voltammograms (S2 Fig. in S1 Appendix) of the modified electrodes show higher capacitance for the OPE and biphenyl SAMs compared to SAMs formed from the C11 alkane which could indicate detects in the SAMs.

**Table 1:** Characteristics of SAMs on gold^a^.

SAM	Contact angles	Coverage, nmol/cm^2^	double layer capacitance, µF/cm^2^
C11	99.5±1.5	0.74±0.11	5±0.5
OPE	74.6±1.5	0.78±0.35	19±2
Biphenyl	84.1±1.1	0.77±0.35	20±2

a average of three electrodes.

### Square wave voltammetry of TNT at SAM modified electrodes

**Figure 2:**
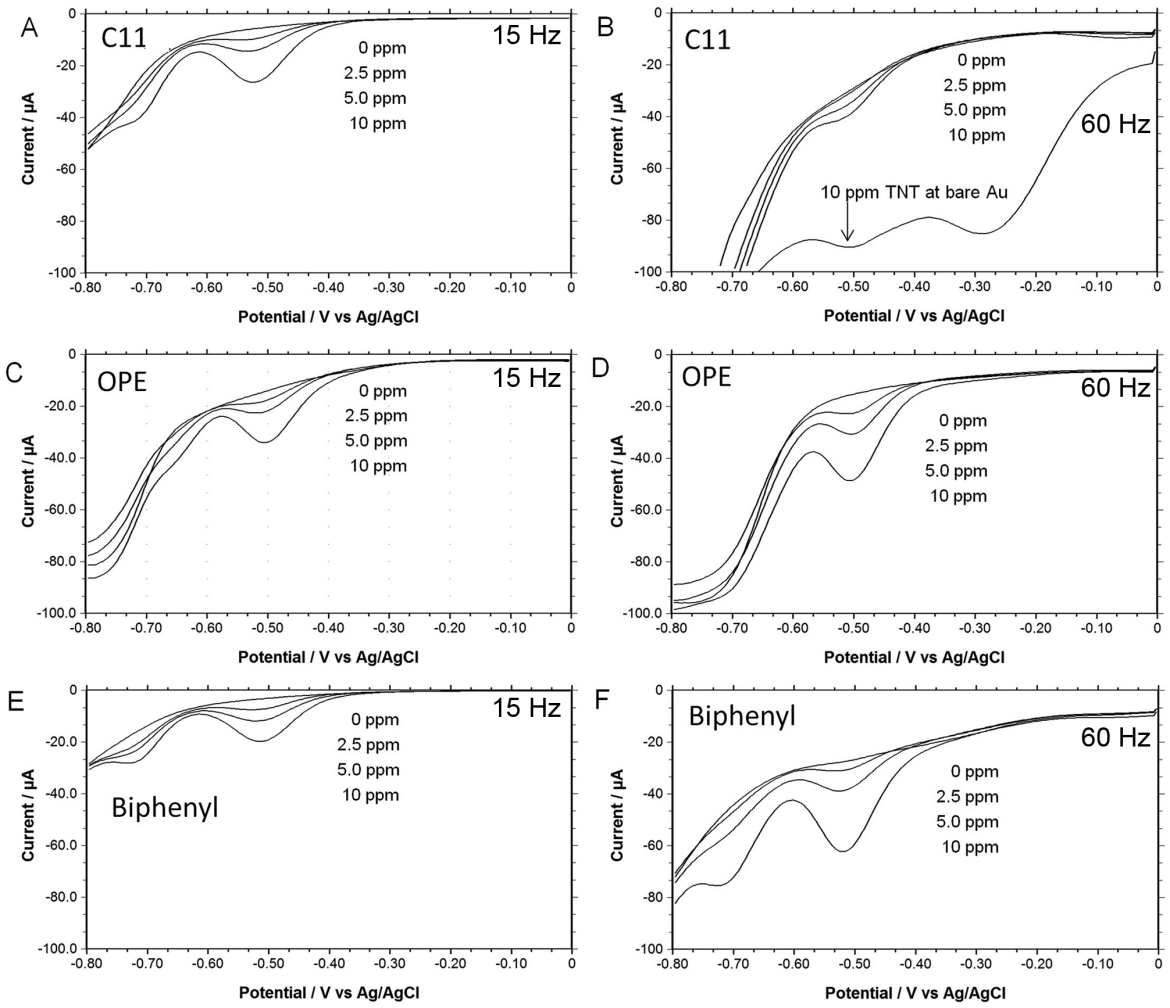
Square wave voltammetry of several TNT concentrations in air-saturated 100 mM potassium phosphate, buffed at pH 8. **A.** The response of TNT at a C11 modified gold electrode measured at 15 Hz. **B.** The response of TNT at a C11 modified gold electrode measured at 60 Hz including a comparison to a bare gold electrode. **C** & **D.** The response of TNT at a OPE modified gold electrode measured at 15 Hz and at 60 Hz, respectively. **E** & **F.** The response of TNT at a Biphenyl modified gold electrode measured at 15 Hz and at 60 Hz, respectively.

To measure the electrochemical response of the reduction of TNT at each of the SAM modified electrodes we recorded square wave voltammograms in a 100 mM potassium phosphate buffer, pH 8. The electrochemistry of TNT has been reported to be independent of pH between 5.5 and 8.5 [5]. Background measurements were recorded before the addition of TNT. Representative examples of the square wave voltammetry for the reduction of TNT at these modified gold electrodes are shown in Fig. 2 measured at 15 and 60 Hz. The square wave voltammograms were measured in air-saturated solutions from 0 to −0.8 V vs. Ag/AgCl. Excessive scans or potentials scanned beyond −0.8 V vs. Ag/AgCl showed clear signs of SAM degradation, most likely due to reductive desorption [8], [9].

The cathodic peak potentials (E_pc_) are listed in Table 2 for the first two reduction peaks of TNT, as recorded in the square wave voltammograms at each SAM and frequency tested. The cathodic peak potentials are similar (between −0.50 to −0.52 V vs. Ag/AgCl) for the 1^st^ reduction of TNT at both 15 Hz and 60 Hz, however, the 2^nd^ reduction at the OPE SAM is significantly less negative by 70 mV when compared to the C11 or biphenyl SAM. Furthermore, at 60 Hz the 2^nd^ reduction is not apparent at the C11 or OPE SAM.

**Table 2:** Electrochemical experimental data for the reduction of TNT^a^.

SAM	1^st^ E_pc,_ V vs. Ag/AgCl		2^nd^ E_pc,_ V vs. Ag/AgCl		Sensitivity, µA/ppm (1^st^ I_pc_)		Limit of detection, ppm (1^st^ I_pc_)^b^	
	15 Hz	60 Hz	15 Hz	60 Hz	15 Hz	60 Hz	15 Hz	60 Hz
C11	−0.52	−0.52	−0.72	-	1.63±0.03	0.96±0.05	0.9	1.6
OPE	−0.50	−0.50	−0.65	-	1.44±0.02	3.16±0.04	0.9	0.4
Biphenyl	−0.52	−0.52	−0.72	−0.72	1.70±0.02	4.1±0.2	1	0.5

1st E_pc_ = the first cathodic peak potential, 2nd E_pc_ = the second cathodic peak potential, 1^st^ I_pc_ = the first cathodic peak current.

a Measured in air saturated water buffered at pH 8 with 100 mM potassium phosphate.

b Limit of detection calculated at S/N = 3.

Additionally, as shown in Fig. 2B, the reduction of TNT at the bare electrode is nearly obscured by the reduction of oxygen in the air-saturated solution, suggesting that the modified gold electrode with the SAMs suppressed the electrochemical activity of the dissolved oxygen. This highlights one of the advantages of modified gold electrode using SAMs.

Partition experiments were conducted in which the SAMs where exposed to 10 ppm solutions of TNT for 5 minutes and then rinsed. These did not show any electrochemical signal of TNT strongly bound to the film (data not shown). We interpret this to mean that our results are not due to any sort of pre-concentration of TNT within the monolayer.

**Figure 3:**
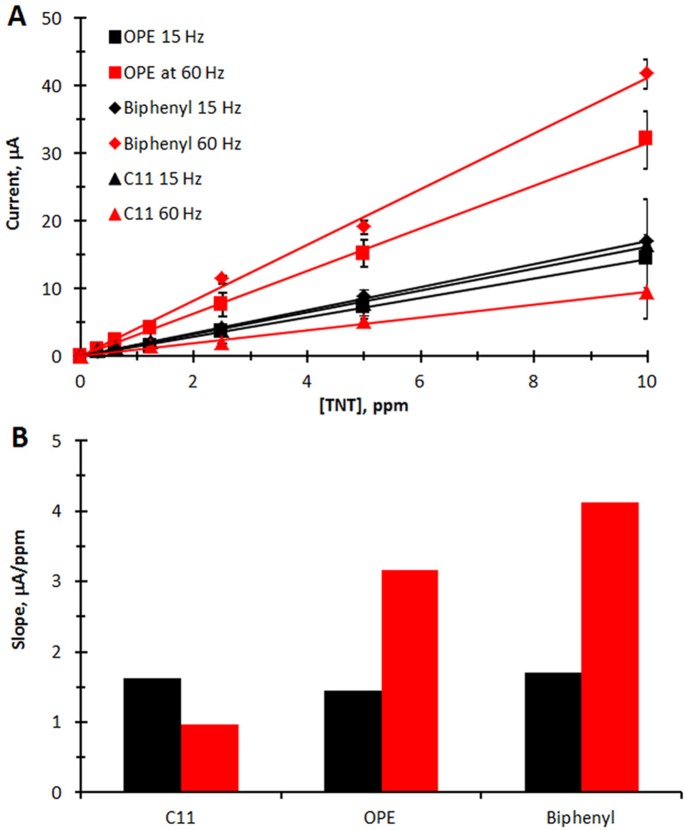
Characteristics of electrochemical detection of TNT. **A.** Background subtracted peak current vs. [TNT] measured at the first reduction of TNT from the corresponding square wave voltammograms (average of two electrodes). **B** Slope (µA/ppm) of the calibration curves vs. SAM type and Hz. Black = 15 Hz, Red = 60 Hz.

The peak currents of the first reduction of TNT show a direct proportionality with concentration between 0.3 and 10 ppm of TNT as shown in Fig. 3A. In Fig. 3B, the slope, i.e. sensitivity (µA/ppm) is also shown for comparison. The sensitivity and limit of detection are also listed in Table 2. At 15 Hz, each SAM gives a slope of about 1.5 µA/ppm. However at 60 Hz, the slope increases for the SAMs containing the aromatic rings, but decreases at the C11 SAM. This is in contrast to carbon electrodes in which the peak currents greatly increase with the frequency up to 50 Hz, and then decay slowly [5].

### Simulations of Square wave voltammetry of TNT

To further characterize and understand the different responses of the SAMs for the reduction of TNT at the different frequencies, we set out to simulate the square wave voltammetry using the electrochemical simulation software DigiElch version 7. The simulation details have been included in S1 Table in S1 Appendix. At each nitro group, reduction occurs and transforms the group to arylhydroxylamines [23], [24]. The signal corresponding to the electrochemical reduction of nitro-aromatics depends on the number of nitro groups on the ring and their relative ring positions. For the reported cyclic voltammetry (CV) studies of 2-nitrotoluene, 3,6-dinitro- toluene, and 2,4,6-trinitrotoluene at carbon electrodes, each peak has been assigned to the reduction of a single NO_2_ group [1]–[3].

From a classical Butler-Volmer simulation, the square wave voltammograms were manually fitted to the experimental data by varying the parameters, *E* (the formal reduction potential) and *k_0_* (the heterogeneous electron transfer rate constant) for each peak. Experimental parameters in the simulation also included the frequency of the square wave in Hz, the concentration the TNT in solution, the diffusion coefficient of TNT (set at typical value of a small molecule in water equal to 1×10^−5 ^cm^2 ^s^−1^) [9] and the geometric area of the electrode. The simulation calculates the forward and reverse Butler-Volmer currents
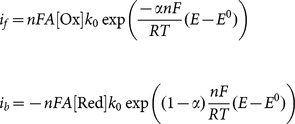



as a function of potential, *E*, where *n* is the number of electrons transferred, *F* is the Faraday constant, *A* is the area of the electrode, [Ox] and [Red] are the concentrations of the oxidized and reduced forms of the substrate, *α* is the transfer coefficient, *E^0^* is the formal potential, *T* is the temperature and *R* is the universal gas constant. The overall current is the sum of these two quantities. In square wave voltammetry the final result is the current difference between successive up-steps and down-steps. Readers wishing more information can consult the standard texts [9], [31].

**Figure 4:**
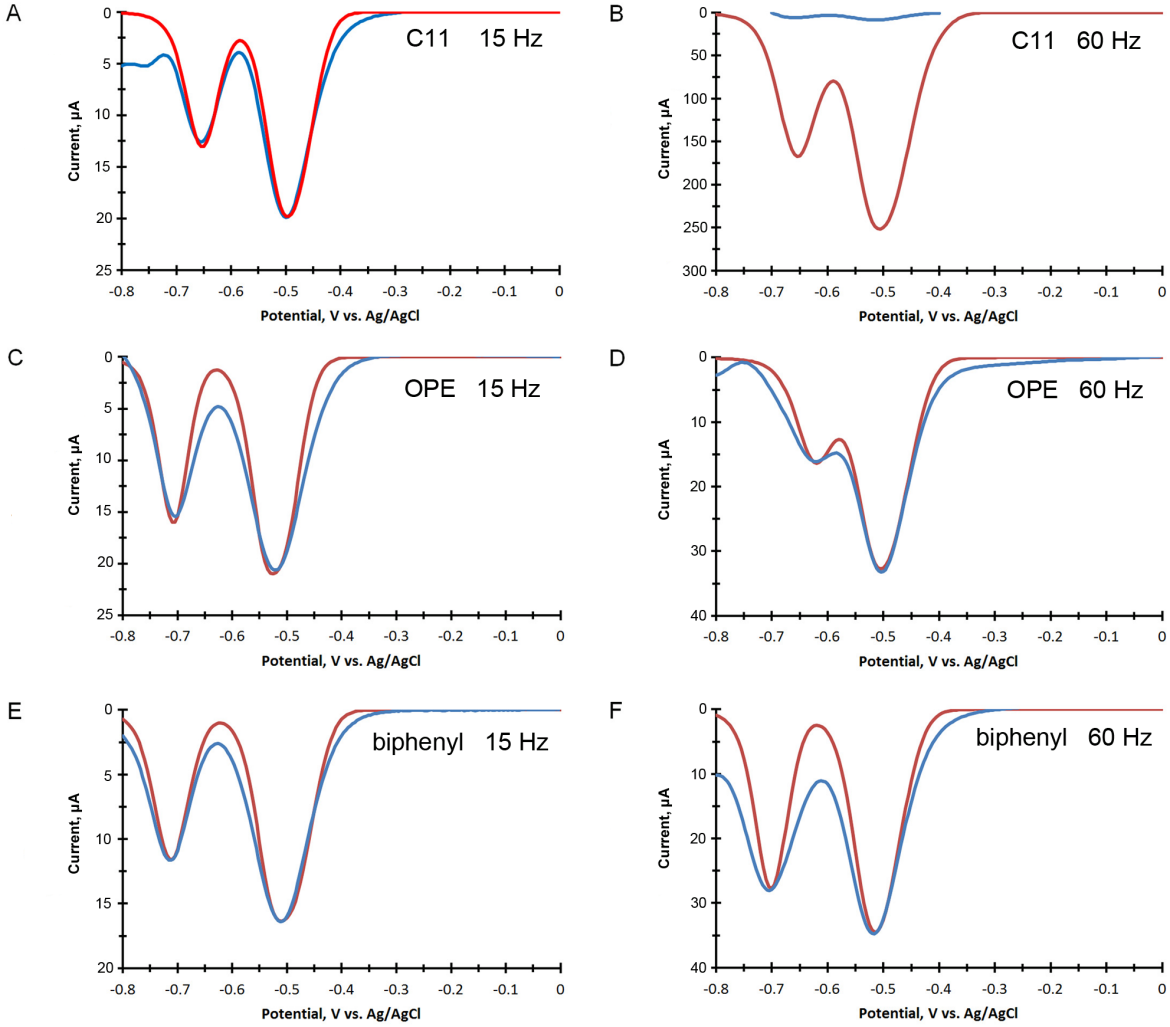
Background subtracted square wave voltammograms of TNT at modified gold electrodes with experimental data (blue trace) compared to simulations (red trace). Fig. 4A and 4B show the response at a C11 SAM at 15 Hz (A) and 60 Hz (B). For 4B the simulation at 60 Hz could not fit the experimental data, and the parameters for the 15 Hz simulation are repeated for comparison. Figs. 4C and 4D shows the response at the OPE SAM at 15 Hz (A) and 60 Hz (B), Figs. 4E and F shows the response at the biphenyl SAM at 15 Hz (A) and 60 Hz (B). Experimental conditions are listed in Fig. 2. Area of the electrode = 0.50 cm^2^, [TNT] = 10 ppm (44 µM). Fitted parameters are listed in Table 2 and simulation settings are listed in the text.

The simulation outputs are listed in Table 3. The direct comparisons of the data (with the background subtracted) to electrochemical simulations are shown in Fig. 4 using a multiple electron transfer model with three 2 electron transfer steps with the electron transfer coefficient set equal to 0.5. The two peaks which are clearly seen in the experimental square wave voltammogram data are included in the analysis. A third reduction peak, often seen at more negative potentials, was not included [1]. At 15 Hz reasonable fits were obtained, but the simulation at 60 Hz with the C11 SAM (Fig. 4B) could not be fit to the experimental data. The data were consistently lower than possible for any realistic combination of *E^0^* and *k_0_*. The red curve in Fig. 4B uses the parameters from Fig. 4A with a frequency of 60 Hz. As can be clearly seen, the experimental data has a much lower than expected magnitude. We suggest that at the higher frequency the blocking nature of the alkane is attenuating the electron transfer between the electrode and TNT, and is doing so in a manner that cannot be accounted for in the Bulter-Volmer model.

**Table 3:** Electrochemical simulation output^a^.

SAM	1^st^ peak, *E*		*k*, cm s^−1^		2nd peak, *E*		*k*, cm s^−1^	
	V vs. Ag/AgCl				V vs. Ag/AgCl			
	15 Hz	60 Hz	15 Hz	60 Hz	15 Hz	60 Hz	15 Hz	60 Hz
C11	−0.46	*-*	0.0040	*-*	−0.50	-	0.004	*-*
OPE	−0.49	−0.46	0.0050	0.0050	−0.53	−0.5	0.0050	0.0050
Biphenyl	−0.46	−0.47	0.0030	0.0053	−0.51	−0.51	0.0030	0.0053

*E* = formal redox potential, *k* = the standard heterogeneous rate constant.

a Each peak was modeled as a 2 electron transfer step. Other parameters are listed in the text.

## Discussion

Square wave voltammetry is sensitive to the rate of electron transfer at the electrode solution interface [9]. For electron transfer rates that are slow compared to the square wave frequency used, the signal will be attenuated. In our examples, when comparing the electrochemical response between simulations and experimental square wave voltammetry data, a general trend was observed in which most of the SAMs had similar heterogeneous rate constants for the reduction of TNT. This observation most likely describes a rate limiting step. However, at higher frequency in the case of the C11 SAM, a decrease in signal was observed which was not consistent with the electron transfer model. It is difficult to explain the data at 60 Hz with the C11 SAM. If the SAM merely resulted in a reduction in the heterogenous rate constant, we would expect to see not a lower magnitude, but a shift to a more negative potential where the larger overpotential would push the effective rate up to a value on the same scale as the other examples in this paper. It may also be the case that the small signal we have measured is due to electron transfer at sites of defective monolayer coverage, and is of low magnitude due to the much smaller effective electrode area. However, we would have expected to be able to discern this by simulation (where it would have revealed itself with a lower *A*, but higher *k*).

Studies on the nature of redox reactions at modified electrodes through the use of SAMs at gold surfaces have provided information on the nature of charge transfer between a redox active headgroup of the SAM and the bridging group used to make the electronic connection to the electrode [25]. For simple one-electron redox couples using bridging groups containing alkanethiol tethers (or saturated bridges), the electron transfer (ET) rate decays exponentially with distance, *k* = *k*_0_exp(−βr), where *k* is the first order ET rate constant measured at a certain distance r (in Å), while *k*_0_ is the ET rate constant at zero distance, and the electron tunneling decay constant, β, is in the range of ∼0.8 to 1 Å^−1^ [26]. In comparison, similar redox couples using delocalized bridges such as oligo(phenylene ethynylene) or oligo(phenylene vinylene) show enhanced electron tunneling with β values ranging from 0.4 to 0.6 Å^−1^, [27], [28] and distance independent redox reaction rates up to 28 Å [29], [30]. We have also discovered similar trends in more complicated redox reactions at modified gold electrodes studying proton-coupled electron transfer for the quinone/hydroquinone redox couple when tethered to gold via similar bridges [12].

In this study, one possible mechanism suggests that the aromatic rings in the SAM are able to tunnel electrons fast enough between the electrode and TNT so that at the higher square wave frequency, the electrochemical signal for the reduction of TNT is not attenuated in the square wave voltammograms. Other factors which may play a role in the difference of the SAMs response at high frequencies include specific interactions with the SAM and the TNT, partitioning of TNT into the SAM and different packing of the molecules in each SAM at the surface of the gold electrode. However, we found no evidence of partitioning of TNT into the SAMs with a strong affinity and the surface coverage of SAMs are similar.

In conclusion the ability to tailor the properties of SAMs by adding aromatic structures at modified electrodes can both increase the response of nitro-aromatics at higher square wave frequency and block the interference of dissolved oxygen. These effects are observed when employing square wave voltammetry by using molecules containing aromatic structures. However, the potential range is limited due to reductive desorption at potentials more negative than −0.8 V vs. Ag/AgCl or with excessive potential sweeps.

## Supporting Information

S1 Appendix Supporting figures and tables. **S1 Fig.** Cathodic stripping voltammetry performed in argon-saturated solutions using 0.5 M KOH buffer at a scan rate of 50 mV/s under Ar for C11 (red), OPE (brown) and biphenyl (blue) SAM modified gold electrodes. Coverages of the SAM molecules on the modified gold electrodes were calculated by integrating the desorption peak at −1.31 V vs. Ag/AgCl for C11, for OPE and biphenyl at −1.09 V vs Ag/AgCl. **S2 Fig.** A. Cyclic voltammograms recorded between 0.2 and −0.2 V vs. Ag/AgCl at a scan rate of 200 mV/s in pH 8 buffer under Ar for C11 (red), OPE (brown) and biphenyl (blue) SAM modified gold electrodes. B. Average of the absolute current at 0 V vs. Ag/AgCl measured from the CVs in A plotted vs. scan rate. Double layer capacitance was calculated from the slope divide by electrode area. **S1 Table.** Additional parameters used by the DigiElch software. Scan parameters are variables used in the simulation of square wave voltammetry. Experimental conditions refer to standardized parameters of the experiment being simulated. Model parameters are a set of variables relating to the computational simulation, and were left in their default conditions.
(DOCX)
