# Supplementary material for: Square Wave Voltammetry of TNT at Gold Electrodes Modified with Self-Assembled Monolayers Containing Aromatic Structures
Source: PLoS One. 2014 Dec 30;9(12):e115966. doi: 10.1371/journal.pone.0115966 (PMC4280194; doi:10.1371/journal.pone.0115966)
Supplement: S1 Appendix — Supporting figures and tables. S1 Fig. Cathodic stripping voltammetry performed in argon-saturated solutions using 0.5 M KOH buffer at a scan rate of 50 mV/s under Ar for C11 (red), OPE (brown) and biphenyl (blue) SAM modified gold electrodes. Coverages of the SAM molecules on the modified gold electrodes were calculated by integrating the desorption peak at −1.31 V vs. Ag/AgCl for C11, for OPE and biphenyl at −1.09 V vs Ag/AgCl. S2 Fig. A. Cyclic voltammograms recorded between 0.2 and −0.2 V vs. Ag/AgCl at a scan rate of 200 mV/s in pH 8 buffer under Ar for C11 (red), OPE (brown) and biphenyl (blue) SAM modified gold electrodes. B. Average of the absolute current at 0 V vs. Ag/AgCl measured from the CVs in A plotted vs. scan rate. Double layer capacitance was calculated from the slope divide by electrode area. S1 Table. Additional parameters used by the DigiElch software. Scan parameters are variables used in the simulation of square wave voltammetry. Experimental conditions refer to standardized parameters of the experiment being simulated. Model parameters are a set of variables relating to the computational simulation, and were left in their default conditions. (DOCX) [file pone.0115966.s001.docx]

**Supporting Information**

**Figure S1.** Cathodic stripping voltammetry performed in argon-saturated solutions using 0.5 M KOH buffer at a scan rate of 50 mV/s under Ar for C11 (red), OPE (brown) and biphenyl (blue) SAM modified gold electrodes. Coverages of the SAM molecules on the modified gold electrodes were calculated by integrating the desorption peak at -1.31 V vs. Ag/AgCl for C11, for OPE and biphenyl at -1.09 V vs Ag/AgCl.

**Figure S2 A.** Cyclic voltammograms recorded between 0.2 and -0.2 V vs. Ag/AgCl at a scan rate of 200 mV/s in pH 8 buffer under Ar for C11 (red), OPE (brown) and biphenyl (blue) SAM modified gold electrodes. **B.** Average of the absolute current at 0 V vs. Ag/AgCl measured from the CVs in **A** plotted vs. scan rate. Double layer capacitance was calculated from the slope divide by electrode area.

**Electrochemical Simulations**

Using DigiElch version 7, the square wave voltammograms were manually fitted to the experimental data by changing the variables, E (the formal reduction potential) and k (the heterogeneous electron transfer rate constant) for the first two peaks in the model listed in equations 1-3 with α = 0.5

A + 2e^-^ → B 1

B + 2e^-^ → C 2

C + 2e^-^ → D 3

Experimental parameters in the simulation included the frequency of the square wave in Hz, the concentration the TNT in solution, the diffusion coefficient of TNT set to 1 x10^-5^ cm^2^ s^-1^ and the geometric area of the electrode. Simulation parameters included a planar geometry with semi-infinite diffusion. Additional parameters used by the DigiElch software are listed in Table A1.

**Table S1** Additional parameters used by the DigiElch software. Scan parameters are variables used in the simulation of square wave voltammetry. Experimental conditions refer to standardized parameters of the experiment being simulated. Model parameters are a set of variables relating to the computational simulation, and were left in their default conditions.

| Scan Parameters | | Experimental Conditions | | Model Parameters | |
| --- | --- | --- | --- | --- | --- |
| dEs (V) | 0.005 | Ru (Ohm) | 0 | Noise Level (%) | 0 |
| Esw (V) | 0.025 | Cdl (F) | 0 | Gauss-Newton Iterations | 1 |
| Time steps/half cycle | 15 | Temperature | 298.2 | Exp. Factor (x-grid) | 0.5 |
|  |  |  |  | Truncation Error (%) | 1E-5 |
|  |  |  |  | Xmax/SQRT(Dt) | 6 |
